# Dihydrolipoamide dehydrogenase deficiency in two unrelated Tunisian children

**DOI:** 10.1186/s12887-024-05375-w

**Published:** 2025-02-22

**Authors:** Hajer Aloulou, Fatma Charfi, Rim Charfi, Amel Ben Chehida, Hela Boudabbous, Imen Chabchoub, Elise Lebigot, Thouraya Kammoun, Ines Maaloul

**Affiliations:** 1 Department of Pediatrics, Hedi Chaker Hospital, Sfax, Tunisia; 2 Faculty of Medecine of Sfax, Sfax, Tunisia; 3 Department of Pediatrics and Inherited Metabolic Diseases, La Rabta Hospital, Tunis, Tunisia; 4 Faculty of Medecine of Tunis, University Tunis El Manar, Tunis, Tunisia; 5 Service de Biochimie-Pharmaco-Toxicologie, Hôpital Bicêtre, APHP-Hôpitaux Universitaires Paris-Saclay, Le Kremlin-Bicêtre, France

**Keywords:** Genetic, Management, E3 deficiency

## Abstract

**Background:**

Dihydrolipoamide dehydrogenase deficiency (DLDD) (OMIM# 246,900) is an extremely rare inherited metabolic disorder causing neurological and/or liver impairment. The clinical manifestations are mostly characterized by severe neurological impairment in early childhood, hepatic presentations and rarely by myopathic manifestations.

**Case presentations:**

Here, we describe two patients presenting with recurrent episodes of vomiting and liver dysfunction. DLDD was confirmed via sanger sequencing by identification of the pathogenic variant c.685G > T (p.Gly229Cys) in DLD gene at a homozygous state.

**Conclusion:**

To our knowledge, this is the first Tunisian report of DLDD. Phenotypic spectrum of this disease is very large. Biochemical markers that predict the impairment of the pathways affected by the deficiency of E3 subunit (gluconeogenesis, tricyclic cycle and catabolism of branched chain aminoacids) are variably present. Confirmation is based on genetic study of DLD gene.

## Introduction

Dihydrolipoamide dehydrogenase (DLD) deficiency is a rare autosomal recessive metabolic disorder caused by mutations in the DLD gene in 7q31.1, encoding for E3 subunit of mitochondrial enzymes (PDH, KGDH, BCKDH). It is a heterogenous disease characterized by the variability of clinical presentations, age at onset and biochemical markers. Clinical manifestations are mostly characterized by severe neurological impairment in early childhood [1, 2], liver involvement [3] and rarely, a myopathic presentation [4].

Early onset DLD deficiency typically manifests in infancy as hypotonia, feeding difficulties, lethargy and occasionally seizures with lactic acidosis. Affected infants frequently die within the first few years of life because of metabolic decompensation. Children, who survive beyond the first two years, present neurological disorders including intellectual disability, spasticity, ataxia and seizures [4].

Isolated liver involvement can present as rarely as the neonatal period and as late as the third decade. It’s preceded by nausea and emesis and frequently associated with encephalopathy [4]. Acute metabolic episodes are triggered by prolonged fasting, inadequate caloric intake and other precipitating factors (infection, catabolic stress, medications) [4, 5].

To date, there is no effective treatment. Prevention of acute episodes is based on special dietary and supplementation with vitamins and coenzymes.

In this article, we describe two unrelated Tunisian children who were diagnosed with DLD deficiency presenting with recurrent episodes of vomiting and liver dysfunction; the diagnosis was based on clinical data and molecular tests.

## Case presentations

### Patient 1

An 11-day-old girl was admitted to our pediatric ward due to vomiting and poor feeding. The child was born at 39 weeks of gestation. APGAR scores were correct (9/10 at 5 and 10 min respectively).

She was the third child of consanguineous parents and had an older sister who died at the age of 8 months after a history of vomiting and neurological deterioration. Her birth weight was 3000 g. The symptoms began the first days after birth, as the child started vomiting daily with an unsatisfactory weight gain. Then, she presented at the age of 11 days with hypo-reactivity and refusal to feed.

At the admission, her weight, length, and head circumference were 2700 g, 51 cm and 33 cm respectively. She was jaundiced, dehydrated, somnolent and hypotonic. Abdominal examination showed hepatomegaly (liver span measured 6 cm). Her capillary blood glucose was 41 mg/dl (Reference values: 50–130). Ketonuria was undetectable in urine.

Laboratory findings revealed hepatic cytolysis (AST/ALT: 660/75UI/l; NV: 8–33), elevated alkaline phosphatase level (687 UI/l; NV: 150–500), normal GGT level (282 UI/l; NV: 23–219), raised total Bilirubin (TB) and conjugated bilirubin (CB) of 183 µmol/l and 28 µmol/l respectively, and a decreased prothrombin time ( 14.7%;NV: 50–70%) with no response to vitamin K injections. The blood ammonia was mildly elevated (69.4 µmol/l; NV:15–60). Acylcarnitine profile, urine organic acids, and plasma amino acids profiles were normal.

The patient received intravenous glucose infusion during 72 h. Vomiting disappeared and liver tests normalized within this period.

The association of recurrent vomiting, acute liver failure in a newborn suggested a congenital galactosemia. The normal activity of the galactose-1-phosphate uridyltransferase (5.6 units/l; normal range: 5—8 units/l) and the negative genetic screening ruled out this hypothesis.

The patient had two other similar attacks respectively at the age of 25 and 31 months. At this time, plasma aminoacids profile was controlled and showed a slight elevation of valine, leucine, and isoleucine. Urine organic acid profile showed an accumulation of lactate (44% of organic acids) and 3-hydroxybutyric acid (5% of organic acids). Gluconeogenesis disorder was suspected and the management consisted of avoiding fasting and fructose/ saccharose loads.

Subsequently, the patient required frequent hospitalizations (4 admissions in one year) with a stereotypical picture of cytolysis (AST/ALT up to 2571/2313 UI/l) with no liver failure, no hepatomegaly nor hypoglycemia. However, the lactate blood concentrations were constantly elevated ranging from 3.5 to 6 mmol/l (reference value: < 2,2).

At 10 years old, the child was admitted for intractable vomiting and excruciating abdominal pain. Despite of the digestive rest and intravenous glucose infusion, the neurological state of the child worsened; she became agitated, confused and had visual hallucinations. Her glycemia and electrolytes were normal. The lactate blood concentration rose to 6.2 mmol/l with hyperammonemia (268 µmol/l).

The patient was then transferred to the pediatric intensive care unit where she was put under glucose infusion (10 mg/kg/minute), oral sodium benzoate (250 mg/kg/day), oral L-carnitine (200 mg/k/day) and intravenous sodium bicarbonate. The child’s health evolved favorably.

**Fig. 1:**
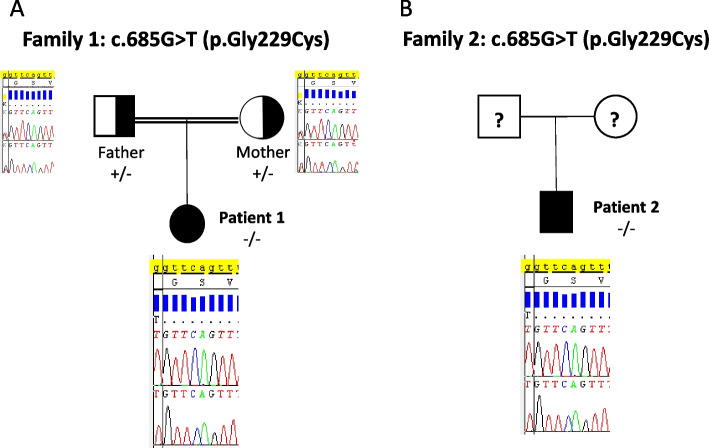
Genotype of both patients for pathogenic variant in *DLD (NM_000108.5:c.685G* > *T; p. (Gly229Cys))*. Sanger sequencing identified that the two patients were homozygous for the same pathogenic variant. The parents of patient 1 were heterozygous carrier for the variant (**A**) while molecular analysis in parents of patient 2 was not performed (**B**)

E3 deficiency was then suspected because of the recurrence of vomiting, abdominal pain, liver dysfunction and the persistence of lactic acidosis. The genetic study of DLD gene coding for dihydrolipoamide deyhydrogenase (protein E3) was performed for the patient. A known pathogenic variant was identified at a homozygous state (NM_000108.5: c. 685G > T or p (Gly229Cys). The parents were heterozygous carrier of this variant (Fig. 1). These findings confirmed the diagnosis of E3 deficiency.

The girl is now 13 years old. She is doing well under special diet (avoiding fasting, uncooked cornstarch 1 g/Kg at bedtime, a slight restriction in protein intake no more than 1 g/kg/day), vitamin B1 (100 mg/day) and L-carnitine supplementation (100 mg/k/day). She still has one to two episodes per year related to skipping her diet or fasting.

### Patient 2

A 9-year-old boy was admitted to our department for vomiting, discomfort and recurrent hypoglycemia.

He was born after a full-term gestation following an uneventful pregnancy. Birth weight was 3100 g and the neonatal period was uneventful.

His parents were healthy and non-consanguineous. Family history showed a female cousin who died at the age of 21 years due to an unexplained neurological deterioration. The symptoms started at the age of 7 years with episodes of weakness, discomfort and unconsciousness.

Hypoglycemia was suspected and the child was admitted for further investigations.

On clinical examination, the child had hepatomegaly with normal anthropometric measurements and capillary glucose level (60 mg/dl;NV:50–130). Initial investigations revealed cytolysis (AST/ALT: 334/530UI/l), liver failure (PT: 35% with no response to vitamin K administration). Plasma ammonia and lactate levels were normal (40 µmol/L and 2.1 mmol/l respectively).

Plasma aminoacids and urinary organic acids profiles were normal, as well as the acylcarnitine profile. A deficiency of neoglucogenesis pathway was suspected. Fructose-1,6- biphosphatase activity was measured in leucocytes and was normal.

The patient received intravenous glucose infusion (8 mg/kg/minute) with electrolytes. The clinical and biological states improved rapidly and the child was discharged.

Three months later, he was readmitted for another episode of severe hypoglycemia with hepatic cytolysis and liver failure. The episode was successfully managed with only glucose infusion.

E3 deficiency was suspected. Molecular analysis of DLD gene was performed by Sanger method. It revealed that the patient was homozygous for the pathogenic variant Gly229Cys confirming our second case of of DLDD (Fig. 1).

Parent’s molecular analysis was not yet performed to confirm biallelic repartition of the identified variants.

Management of the disease was based on avoiding fasting, supplementation with thiamine (100 mg/day) and L-carnitine(100 mg/kg/day). No other acute metabolic decompensation was observed after 3 years of follow up.

## Discussion

Dihydrolipoamide dehydrogenase deficiency is an extremely rare autosomal recessive metabolic disorder caused by mutations in the *DLD* gene in 7q31.1 [4]. Until 2021, 40 individuals were reported in the literature [4]. Since this date, some case reports or case series were reported [6–8]. To our knowledge, these are the first two cases of DLD deficiency reported in Tunisia.

DLD functions as the E3 subunit of three mitochondrial enzyme complexes: branched-chain alpha-ketoacid dehydrogenase (BCKDH) complex, α-ketoglutarate dehydrogenase (αKGDH) complex, and pyruvate dehydrogenase (PDH) complex [4, 9]. The E3 subunit is responsible for the reoxidation of the reduced lipoyl moiety of the E2 subunit of these enzymes.

The phenotype spectrum of the disorder is very heterogenous. The majority of patients presented with neurological impairment associated to neonatal lactic acidosis or later episodic acute metabolic decompensation with elevated lactate. Neurologic involvement consisted frequently of delayed development and hypotonia during infancy. Severe encephalopathy was observed in case of an inadequate care or a delayed diagnosis. Seizures could also be associated [9]. Few patients presented later in childhood with ataxia, dystonia and normal cognitive development [4, 9].

A separate group of patients presented with extra neurological symptoms. They mainly consisted of episodic vomiting, abdominal pain, encephalopathy and liver failure, which was the case of the two patients reported here. According to Quinonez et al. [4], individuals with the hepatic form, present recurrent episodes of liver dysfunction which are frequently preceded by nausea and emesis. Recurrent attacks of hepatopathy are often triggered by dietary extremes (high fat diet, or fasting) or intercurrent illness or fever or some medications. These forms are not associated with neurological defect between acute metabolic episodes and affected children have normal intellect. To date, our patients had normal neurological examination and no intellectual disability.

Affected patients with hepatic presentation can develop symptoms as early as the neonatal period as late as the third decade of life [5]. In our study, both patients presented with an hepatic form associated with few neurological symptoms in patient 1; this latter started in the neonatal period while the other during childhood. Both had the association of cytolysis and/or liver failure and hypoglycemia.

Other symptoms were reported and can include failure to thrive, developmental delay, hepatomegaly, hypoglycemia, hypertrophic cardiomyopathy, isolated lactic acidosis episodes, hyperammonemia among many other manifestations [10].

The diagnosis of DLDD is based on suggestive clinical symptoms with supportive biochemical findings related to pyruvate and aminoacid metabolism.

In the hepatic form of DLDD, laboratory findings can show hepatic cytolysis (isolated elevated transaminases to fulminant hepatic failure), coagulopathy, hypoglycemia, hyperammonemia, elevation of the plasma lactate, elevation of the branched-chain amino acids like leucine, isoleucine and valine, elevation of the urinary lactate and organic acid levels such as alpha-ketoglutarate [4]. Increased plasma citrulline level associated with metabolic acidosis could suggest DLDD [11]. However, these biochemical changes can be absent or intermittent [12], as it was the case in both our patients.

In addition, diagnosis can be established by decreased enzymatic activity in lymphocytes or fibroblasts and confirmed by molecular genetic testing through the identification of biallelic pathogenic variants in *DLD*.

The common c.685G > T variant of the *DLD* gene (p.Gly229Cys) was initially described in the middle east Ashkenazi Jews and Palestinian Arabs presenting with isolated liver involvement [3, 13]. Then it was reported in the Maghreb, in five members of an Algerian family [5], and now it is described in Tunisia after we identified this mutation in two unrelated cases who were indeed found to be homozygous for this variant.

Phenotypic severity is difficult to predict based on genotype; however, some correlations have been reported for individuals who have c.685G > T pathogenic variant; in fact, all patients with exclusively hepatic presentation like patient 2, have been homozygous for c.685G > T variant [5]. This variant was found in five patients (62%) with DLDD in a Saudi study and was responsible mostly for hepatic form; only 2/5 patients had associated psychomotor dysfuntion [14].

Guidelines for the management of DLDD does not currently exist. In a summary published by Quinonez and al in 2021 [4], some recommendations for the management of the disease were mentioned. In fact, acute treatment in individuals with acute liver injury or failure due to DLDD includes: treatment of any precipitating factors (infection, fasting, medications), dextrose-containing IV fluids (6–8 mg/kg/min) with age-appropriate electrolytes and/or frequent feedings. The treatment also includes correction of lactic acidosis, using sodium bicarbonate for severe metabolic acidosis (pH < 7.20 or bicarbonates < 14 mEq/L), considering dichloroacetate supplementation at a daily dose of 50 to 75 mg/kg and dialysis if persistent lactic acidosis or encephalopathy. Fresh frozen plasma can be administered if coagulopathy [4].

Limited data exist for chronic management of individuals with the primarily hepatic presentation. Between episodes, affected individuals typically return to baseline and do not require treatment beyond avoidance fasting, catabolic stressors and liver-toxic medications [4]. Moreover, supplementation of levocarnitine at a daily dose of 75–100 mg/kg and thiamine is recommended [4].

## Conclusion

The diagnosis of DLDD can be challenging especially in the absence of biochemical clues and in the cases of isolated hepatic impairment. Physicians should think about DLD deficiency in children presenting with recurrent episodes of liver dysfunction. Targeted analysis for the c.685G > T (p.Gly229Cys) pathogenic variant should be considered first in patients presenting recurrent episodes of liver dysfunction/failure, especially in patients from a Mediterranean country.

## Data Availability

The OMIM accession number for the sequence of the DLD gene is available on the database (https://www.omim.org/entry/246900).

## Abbreviations

ALT: Alanine aminotransferase
APGAR: Appearance, pulse, grimace, activity and respiration
AST: Aspartate aminotransferase
BCKDH: Branched-chain alpha-ketoacid dehydrogenase
CB: Conjugated bilirubin
DLDD: Dihydrolipoamide dehydrogenase deficiency
α KGDH: α-Ketoglutarate dehydrogenase
PDH: Pyruvate dehydrogenase
PT: Prothrombin time
TB: Total bilirubin
IV: Intravenous
NV: Normal values
